# The Scope of Physiotherapy Rehabilitation in Compressive Myelopathy Managed by Spinal Fusion: A Case Report

**DOI:** 10.7759/cureus.48290

**Published:** 2023-11-05

**Authors:** Ghanishtha Burile, Swapna Jawade, Nikita Seth

**Affiliations:** 1 Department of Musculoskeletal Physiotherapy, Ravi Nair Physiotherapy College, Datta Meghe Institute of Higher Education and Research, Wardha, IND

**Keywords:** decompression, hypertrophy of ligamentum flavum, functional limitation, quality of life, spinal fusion, physical therapy, spondylosis, cervical myelopathy

## Abstract

Cervical myelopathy is a sequence of alterations that cause etiological ailments such as spondylosis, ossification of the posterior longitudinal ligament, and compression of nerve roots at various levels. The reduced diameter of the vertebral canal is because of degenerative changes in the structure of the disc, along with the formation of osteophytic spurs that compress the surrounding structures, such as nerve roots, at one or more levels. Radiography, CT, MRI, and dynamic study help identify cervical spondylotic myelopathy. Surgical methods such as anterior, posterior, or combined approaches are used to stabilize and potentially improve the subject’s neurologic status. The spine’s alignment, the number of mobility segments implicated, the morphology, and the location of the spondylotic compression guide surgical decision-making. Cervical spondylotic myelopathy is a condition of the cervical spine that causes narrowing of the spinal canal with symptoms such as neck pain, numbness in the hands, gait problems, and sphincter dysfunction. We present the case of a 52-year-old male diagnosed with compressive myelopathy from C3 to C7 with a history of falling from the bed. On MRI, there were degenerative changes, spondylosis, and compressive myelopathy, and a disc bulge at multiple levels was seen. The patient underwent a spinal fusion at C3 to C7 level followed by structured physical therapy rehabilitation to gain a good recovery and functional independence to improve quality of life.

## Introduction

Cervical spondylotic myelopathy, or degenerative cervical myelopathy, is a common serious neurological disorder in adults. The etiology of the condition could be compression at the spinal cord level. Common symptoms include neck pain, functional limitation, and motor functions in arms, fingers, and hands. If the patient does not take proper treatment, it could lead to permanent nerve damage and paralysis [1]. Posterior decompression enlarges the space needed for the spinal cord and permits the cord to move backward from the front structure. The benefits of the posterior approach include the decompression of nerve under imagination and prevention of injury to anterior structures along with some major vessels, the esophagus, and recurrent laryngeal nerve [2].

Static and dynamic stressors can lead to degenerative cervical myelopathy. There is stenosis of the developmental canal, intervertebral disc bulging, along with hypertrophy of ligamentum flavum. Dynamic stressors include the invagination of the ligamentum flavum [3]. Posterior decompression and fusion and laminoplasty are used to treat cervical myelopathy due to multilevel ossification of the posterior longitudinal ligament [4]. In the surgical method of posterior decompression in spondylotic myelopathy of the spine, a recovery rate of 30% to 55% has been reported [5]. Overall, 21% of cervical myelopathy patients fail to exhibit any myelopathic indications, indicating that myelopathic symptoms were not very sensitive in detecting the existence of cervical myelopathy [6]. The pathophysiology of degenerative compression is apoptosis of cellular structures, vascular changes, inflammatory responses, axon degeneration, and myelin changes. Wallerian degeneration in the white matter of motor axons in the lateral corticospinal tract leads to clinical symptoms such as spastic gait [7].

In patients with anterior cervical decompression, strength increases by 80 to 90% in each muscle group. Patients feel functional improvements in their lower extremities; sometimes, there is a discrepancy due to prolonged spasticity rather than muscle weakness [8]. The etiology of the condition could be due to spinal cord dysfunction among adults aged 55 years, along with risk factors causing traumatic central cord syndrome, the most common cause of cervical spinal cord injury [9]. Assessment findings include stiffness in the neck region, a wide-base ataxic gait, ascending paraesthesia in both extremities, lower extremity weakness, reduced hand dexterity, hyperreflexia, clonus, Babinski sign, and bowel or bladder dysfunction [10]. In all, cervical myelopathy is diagnosed in 18.1% of patients nationwide receiving cervical decompression myelopathy. Compared to individuals without myelopathy, patients receiving cervical decompression myelopathy are more likely to be older [11]. Other symptoms of cervical degeneration include cervicogenic headaches, vertebrobasilar symptoms, and precordial pain [12]. However, the chord’s transverse area at the greatest compression level offers the most accurate and complete criterion for their prognosis. Various factors, including the chronicity of the disease and age at surgery, are thought to impact the outcomes of the surgery [13].

Cognitive impairments can be useful in differentiating normal pressure hydrocephalus from cervical spondylotic myelopathy [14]. A cervical collar for a minimum of eight hours regularly for three months helps decrease the duration of physical therapy rehabilitation, along with rest or performing activities of daily living. Physical therapies can be preferred for pain reduction which include massage, spine ice, transcutaneous electrical nerve stimulation, and ultrasound. Exercises prescribed for strengthening or stretching the muscles of the upper back, shoulder, and upper extremities, as well as aerobic exercises, increase the oxygen intake of patients. Guidance about ergonomic and postural alignment management can be advised to prevent complications, and slight traction and mobilization of the cervical spine can be performed [15].

## Case presentation

A 52-year-old male with right-hand dominance had an accident with a fall from a bed of approximately 2 feet in height while trying to wake up in August 2023. After the fall, the patient was conscious and well-oriented, but there was weakness in both lower limbs and upper extremities. Immediately after the fall, the patient was taken to a local hospital. Investigations such as an MRI of the brain did not show any significant findings. An MRI of the cervical spine revealed edema/myelomalacia at the C3 to C7 level.

The patient was referred to the tertiary care hospital on August 12, 2023, and advised for spinal fusion. The patient was operated on August 18, 2023, at the C3-C7 level, and lateral mass screws were inserted at C3, C4 right side, and C4 left side. Decompression (laminectomy) was done at C3-C7, and hypertrophied ligamentum flavum was removed. The patient complained of difficulty lifting both lower extremities and limited upper extremity movements. As mentioned in Table 1, the reflexes of joints were assessed. Hyperreflexia was noted in the lower body and upper extremities below the lesion level. Using manual muscle testing numbness, paraesthesia in one or both hands, along with radiculopathy signs were seen (Table 2). Physiotherapy rehabilitation was initiated on the first postoperative day after a thorough assessment, which revealed distal weakness, weakness in distal extremities such as the hand, and difficulty in performing fine motor movements. The outcome measures used were the Numerical Pain Rating Scale, manual muscle testing, and Cooper myelopathy (Table 3).

**Table 1:** Reflexes.

Reflexes	Right	Left
Biceps reflex	+++	+++
Triceps reflex	+++	+++
Brachioradialis reflex	++	++
Patellar reflex	+	+
Achilles reflex	0	0

**Table 2:** Manual muscle testing (MMT).

Joints	MMT grade (right)	MMT grade (left)
Shoulder flexors	1	1
Shoulder extensors	1	1
Elbow flexors	1	1
Elbow extensors	1	1
Wrist flexors	1	1
Wrist extensors	1	1
Hip flexors	0	0
Hip extensors	0	0
Knee flexors	0	0
Knee extensors	0	0
Ankle plantar flexors	0	0
Ankle dorsiflexors	0	0

**Table 3:** Outcome measures.

Outcome measures	Four weeks	Six weeks
Numerical Pain Rating Scale	On activity: 6/10; on rest: 4/10	On activity: 4/10; on rest: 3/10
Manual muscle testing (postoperative)	Right	Left
Shoulder flexors	1	1
Shoulder extensors	1	1
Shoulder abductors	1	1
Elbow flexors	1	1
Elbow extensors	1	1
Wrist flexors	1	1
Wrist extensors	1	1
Hip flexors	1	1
Hip extensors	1	1
Knee flexors	1	1
Knee extensors	1	1
Ankle plantar flexors	1	1
Ankle dorsiflexion	1	1
Cooper myelopathy scale		
Preoperative	Cooper myelopathy scale (Grade 4): Little movement but cannot walk/stand	Cooper myelopathy scale (Grade 5): Paralysis
Postoperative	Cooper myelopathy scale (Grade 2): Walks but needs a cane or a walker	Cooper myelopathy scale (Grade 3): Stands but cannot walk

Investigations

**Figure 1:**
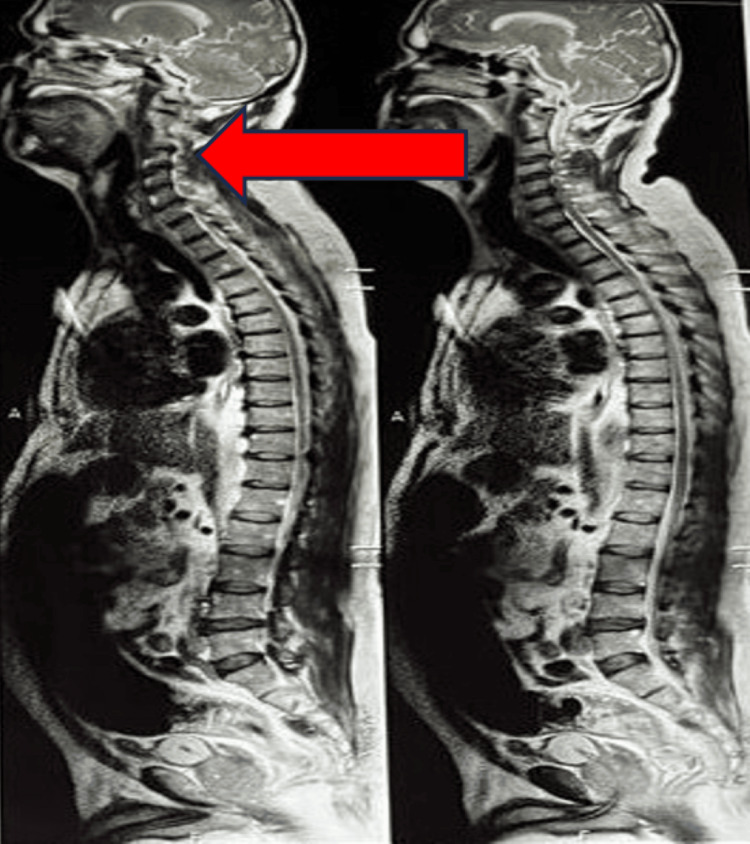
MRI of the cervical spine reveals compressive myelopathy with spondylosis (red arrow).

MRI of the cervical spine revealed compressive myelopathy with spondylosis, as indicated by the red arrow in Figure 1.

Physiotherapy rehabilitation protocol

The patient was given physical therapy rehabilitation for six days a week for 50 minutes per day. The protocol to be followed using a timeline of the rehabilitation is presented in Table 4.

**Table 4:** Physiotherapy rehabilitation protocol.

Problem	Goal	Intervention	Rationale
Lack of proper knowledge of the condition may increase the risk of complications	To prevent complications and risk factors	Explain the condition to the patient, along with the precautions and benefits of physical therapy rehabilitation in recovery	To make the patient independent and improve the quality of life
Decrease the range of motion (ROM) of the upper extremity	To improve the ROM of the upper extremity	Active assisted exercises of the upper extremity	Performed due to reduced strength
Reduced cervical ROM	To maintain the stability of the cervical spine	Cervical collar	It stabilizes the weight of the head while the bone, soft tissues, and muscles in the neck are healing
Difficulty in coughing and swallowing	To aid in removing secretions	Thoracic expansion exercises	Helps in improving chest mobility and aids in the clearance of secretions
Difficulty in breathing.	To reduce the work of breathing.	Deep breathing exercises	Decrease the occurrence and severity of pulmonary complications, such as pneumonia, atelectasis, along with hypoxemia
Difficulty in initiating movement of the ankle	To make the patient initiate movement voluntarily	Rood’s approach of icing using the plantar grasp reflex to gain knee flexion	Proprioceptors in a single segment of the kinetic chain should function adequately for proper movement patterns to happen. Proprioceptive input for motor control
The patient is unable to stand	To make the patient stand independently	Tilt table and a standing frame	Helps in adaptation to the antigravity position
Posture affected	To maintain good postural alignment	A proper pillow is placed under the neck, upper extremity, and lower extremity	Reduces back pain, increases energy, and improves confidence
Orthostatic hypotension due to prolonged bed rest	To decrease the symptoms of light-headedness or dizziness when sitting	Head up tilt during sleep. Increase in fluid intake. Use of a tilt table	The Tilt table provides a platform to increase the adaptation of the body against gravity
Decreased motor functions	Improve motor function recovery	Transcranial magnetic stimulation combined with physiotherapy [16]	Produces changes in cortical activity

**Figure 2:**
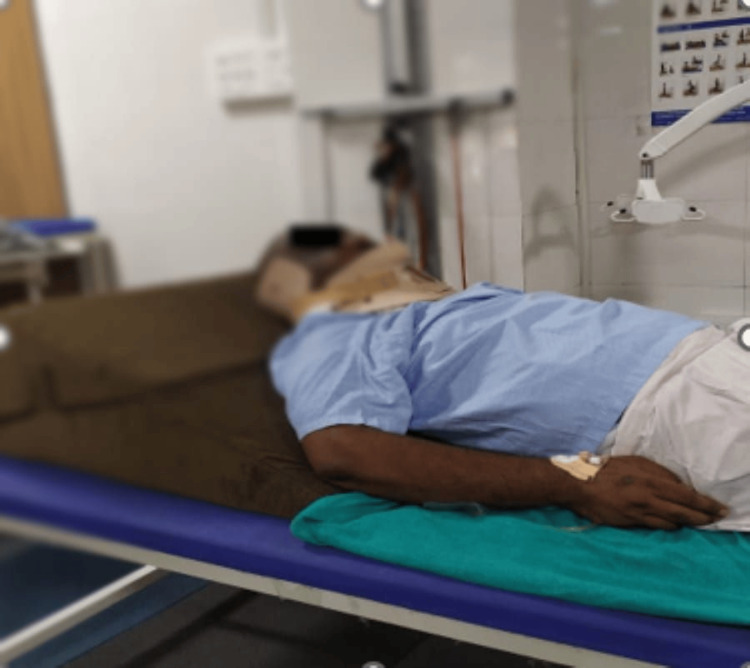
Patient’s head elevated to decrease the symptoms of orthostatic hypotension.

**Figure 3:**
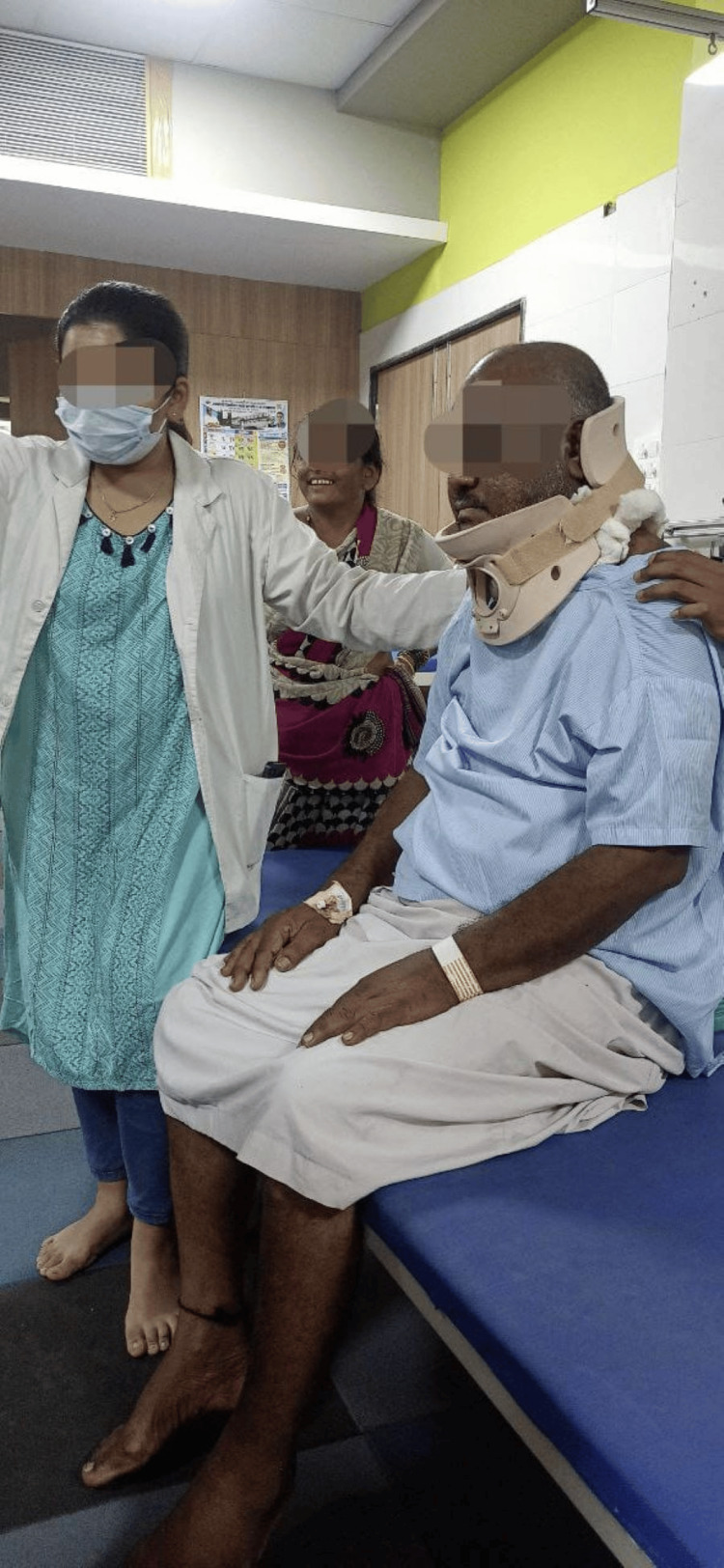
The patient being assisted while sitting by the therapist.

**Figure 4:**
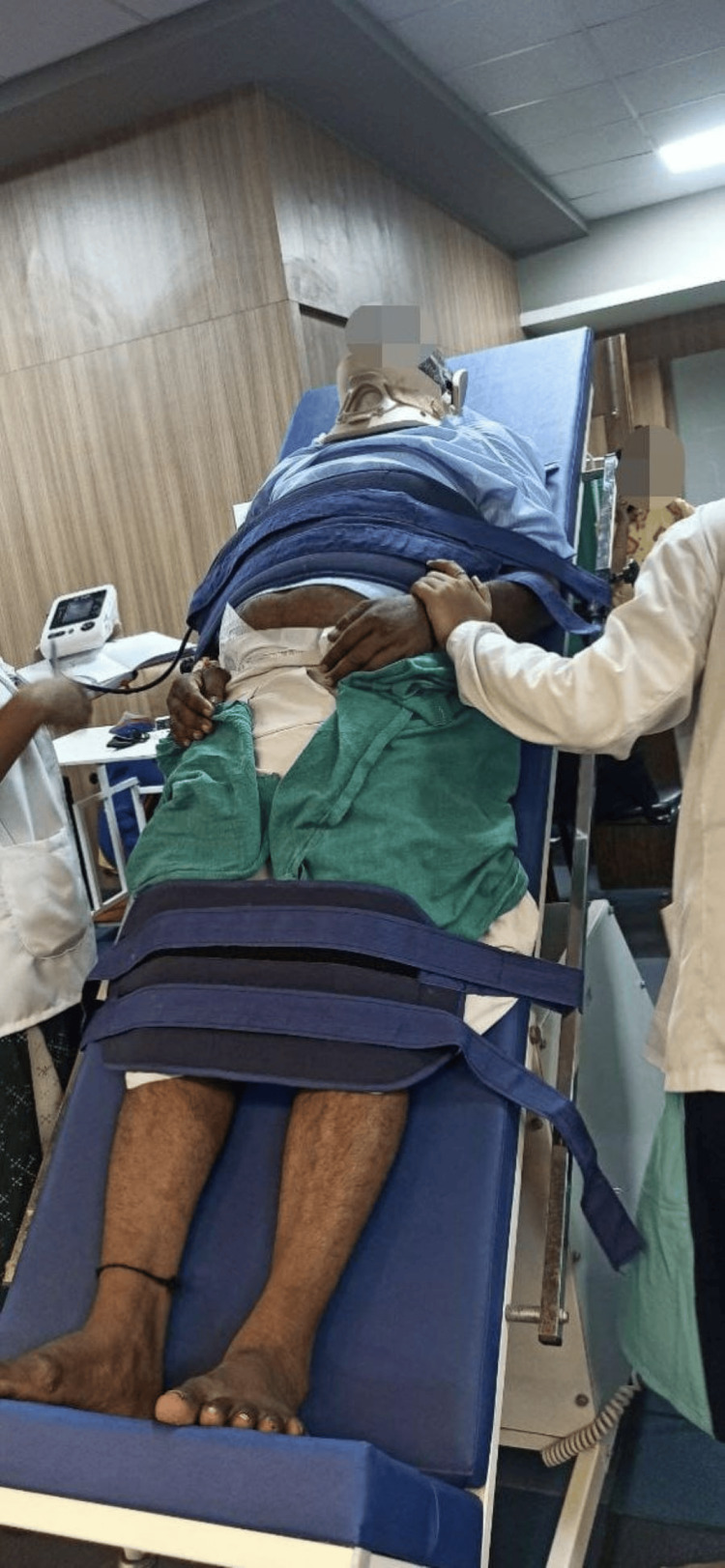
The patient placed on a tilt table to reduce the symptoms of orthostatic symptoms.

A customized rehabilitation plan was created for the patient. As shown in Figure 2, the patient’s head was elevated to decrease the symptoms of orthostatic hypotension. The patient was assisted while sitting by the therapist (Figure 3). The patient was placed on a tilt table to reduce the symptoms of orthostatic symptoms (Figure 4).

## Discussion

Nowadays, lateral mass screw fixation with bone grafts provides good functional recovery. Laminectomy and fusion can lead to non-union, hardware failure, segment degeneration, absent lordosis, and irritation at the autograft harvest site [17]. Nowadays, the most commonly used treatment in cervical myelopathy is the surgical method, in which the cord decompression and spine are supported. Surgical approaches can involve anterior or posterior approaches [18]. For less distance of about <4 mm, an anterior approach or combined anterior/posterior approach is associated with generating good postoperative outcomes compared to the posterior approach [19]. The patient in this report underwent structured physical therapy rehabilitation with many exercises from a qualified orthopedic physiotherapist. Using a tilt table causes a gradual decrease in symptoms such as vertigo and loss of balance, enabling the patient to exert more effort during rehabilitation and gradually improving postural alignment, range of motion, and functional outcomes.

Physical therapy aimed to avoid neurological complications and bed sore in the lower back as there was no movement in both lower extremities. The use of a cervical collar causes premature occurrence of the condition. In patients with shorter illness durations and minimal cord atrophy in the neutral neck position, improvement is anticipated [20]. Surgery can decrease complications, and early surgery has good outcomes. There is a need to care for patients with mild symptoms. The goal of the above case study was to highlight the significance of essential physiotherapy rehabilitation and the spinal fusion program in achieving functional objectives for the patient in the long term.

## Conclusions

Conservative treatments for cervical spondylotic myelopathy include neck immobilization, pharmacologic interventions, changes in diet, proper exercise schedules, and physical therapy modalities. The tailored physiotherapy protocol should be followed before any surgical procedure to achieve better results after surgery. An early indication of an emergency operation would be a complete weakness in all four limbs. More high-level studies comparing these modalities of surgical interventions are needed. This patient was treated surgically with spinal fusion. Rehabilitation protocols should be thoroughly grounded and individually tailored for the best outcomes. However, common ways to help with this illness include boosting muscular strength, enhancing the range of passive or active movement, and promoting and preventing further functional losses as the condition worsens. To ensure the finest therapeutic practices in cervical spondylotic myelopathy such therapeutic methods should be recorded to make them accessible to other studies and clinical practice.

## References

[1] Tu J, Vargas Castillo J, Das A, Diwan AD (2021). Degenerative cervical myelopathy: insights into its pathobiology and molecular mechanisms. J Clin Med.

[2] Koh EY, Ludwig S (2014). Cervical myelopathy: posterior decompression and fusion. Semin Spine Surg.

[3] Baptiste DC, Fehlings MG (2006). Pathophysiology of cervical myelopathy. Spine J.

[4] Xu P, Sun GD, Xun L, Huang SS, Li ZZ (2021). Posterior decompression and fusion versus laminoplasty for cervical ossification of posterior longitudinal ligament: a systematic review and meta-analysis. Neurosurg Rev.

[5] Kawakami M, Tamaki T, Iwasaki H, Yoshida M, Ando M, Yamada H (2000). A comparative study of surgical approaches for cervical compressive myelopathy. Clin Orthop Relat Res.

[6] Rhee JM, Heflin JA, Hamasaki T, Freedman B (2009). Prevalence of physical signs in cervical myelopathy: a prospective, controlled study. Spine (Phila Pa 1976).

[7] Akter F, Yu X, Qin X, Yao S, Nikrouz P, Syed YA, Kotter M (2020). The pathophysiology of degenerative cervical myelopathy and the physiology of recovery following decompression. Front Neurosci.

[8] Chiles BW 3rd, Leonard MA, Choudhri HF, Cooper PR (1999). Cervical spondylotic myelopathy: patterns of neurological deficit and recovery after anterior cervical decompression. Neurosurgery.

[9] Karadimas SK, Moon ES, Yu WR, Satkunendrarajah K, Kallitsis JK, Gatzounis G, Fehlings MG (2013). A novel experimental model of cervical spondylotic myelopathy (CSM) to facilitate translational research. Neurobiol Dis.

[10] Kane SF, Abadie KV, Willson A (2020). Degenerative cervical myelopathy: recognition and management. Am Fam Physician.

[11] Stevenson T, Hart D, Piver M, Ryu W, Yoo J, Orina J (2023). Cervical disc arthroplasty in the treatment of myelopathy: clinical predictors, utilization, and outcomes in a national administrative database. J Spine.

[12] Gibson J, Nouri A, Krueger B, Lakomkin N, Nasser R, Gimbel D, Cheng J (2018). Degenerative cervical myelopathy: a clinical review. Yale J Biol Med.

[13] Fujiwara K, Yonenobu K, Ebara S, Yamashita K, Ono K (1989). The prognosis of surgery for cervical compression myelopathy. An analysis of the factors involved. J Bone Joint Surg Br.

[14] Bakhsheshian J, Mehta VA, Liu JC (2017). Current diagnosis and management of cervical spondylotic myelopathy. Global Spine J.

[15] Oliveira RA, Fares K, Schiavon D, Souza PC, Luvizutto GJ, Souza LAPS (2019). Cervical spondylotic myelopathy: clinical cases and physiotherapy. Coluna/Columna.

[16] Farrokhi MR, Salehi S, Nejabat N, Safdari M, Ramezani Abadeh H (2022). Beneficial effect of repetitive transcranial magnetic stimulation combined with physiotherapy after cervical spondylotic myelopathy surgery. J Clin Neurophysiol.

[17] Choi SH, Kang CN (2020). Degenerative cervical myelopathy: pathophysiology and current treatment strategies. Asian Spine J.

[18] Stoner KE, Abode-Iyamah KO, Fredericks DC, Viljoen S, Howard MA, Grosland NM (2020). A comprehensive finite element model of surgical treatment for cervical myelopathy. Clin Biomech (Bristol, Avon).

[19] Wilson JR, Badhiwala JH, Moghaddamjou A, Martin AR, Fehlings MG (2019). Degenerative cervical myelopathy; a review of the latest advances and future directions in management. Neurospine.

[20] Tokumaru Y, Hirayama K (2001). [Cervical collar therapy for juvenile muscular atrophy of distal upper extremity (Hirayama disease): results from 38 cases]. Rinsho Shinkeigaku.

